# Intracellular Trafficking of the Amyloid β-Protein Precursor (APP) Regulated by Novel Function of X11-Like

**DOI:** 10.1371/journal.pone.0022108

**Published:** 2011-07-19

**Authors:** Yuhki Saito, Mayu Akiyama, Yoichi Araki, Akio Sumioka, Maki Shiono, Hidenori Taru, Tadashi Nakaya, Tohru Yamamoto, Toshiharu Suzuki

**Affiliations:** 1 Laboratory of Neuroscience, Graduate School of Pharmaceutical Science, Hokkaido University, Sapporo, Japan; 2 Laboratory of Neuronal Cell Biology, Graduate School of Pharmaceutical Science, Hokkaido University, Sapporo, Japan; 3 Creative Research Institute Sousei, Hokkaido University, Sapporo, Japan; National Institute on Aging Intramural Research Program, United States of America

## Abstract

**Background:**

Amyloid β (Aβ), a causative peptide of Alzheimer's disease, is generated by intracellular metabolism of amyloid β-protein precursor (APP). In general, mature APP (mAPP, *N*- and *O-*glycosylated form) is subject to successive cleavages by α- or β-, and γ-secretases in the late protein secretory pathway and/or at plasma membrane, while immature APP (imAPP, *N-*glycosylated form) locates in the early secretory pathway such as endoplasmic reticulum or *cis*-Golgi, in which imAPP is not subject to metabolic cleavages. X11-like (X11L) is a neural adaptor protein composed of a phosphotyrosine-binding (PTB) and two C-terminal PDZ domains. X11L suppresses amyloidogenic cleavage of mAPP by direct binding of X11L through its PTB domain, thereby generation of Aβ lowers. X11L expresses another function in the regulation of intracellular APP trafficking.

**Methodology:**

In order to analyze novel function of X11L in intracellular trafficking of APP, we performed a functional dissection of X11L. Using cells expressing various domain-deleted X11L mutants, intracellular APP trafficking was examined along with analysis of APP metabolism including maturation (*O-*glycosylation), processing and localization of APP.

**Conclusions:**

X11L accumulates imAPP into the early secretory pathway by mediation of its C-terminal PDZ domains, without being bound to imAPP directly. With this novel function, X11L suppresses overall APP metabolism and results in further suppression of Aβ generation. Interestingly some of the accumulated imAPP in the early secretory pathway are likely to appear on plasma membrane by unidentified mechanism. Trafficking of imAPP to plasma membrane is observed in other X11 family proteins, X11 and X11L2, but not in other APP-binding partners such as FE65 and JIP1. It is herein clear that respective functional domains of X11L regulate APP metabolism at multiple steps in intracellular protein secretory pathways.

## Introduction

X11 proteins (X11s) comprise a family of evolutionarily conserved molecules, which include: X11/X11α/mint1, X11L/X11β/mint2 and X11L2/X11γ/mint3 in mammals [1], two orthologous proteins in *D. melanogaster* [2], [3], and one in *C. elegans* [4]. X11s are composed of a relatively unique N-terminal half and a conserved C-terminal half, which includes a phosphotyrosine binding/phosphotyrosine interaction (PTB/PI) domain and two PDZ domains. Through protein-protein interactions, mediated by the PTB/PI and/or PDZ domains, X11s are thought to play various important regulatory roles in synapse formation, protein transport and protein metabolism [5].

In mammals, X11 and X11L are expressed predominantly in the brain, while X11L2 is expressed ubiquitously [5]. The PTB/PI domain of X11s binds to the cytoplasmic domain of Alzheimer's amyloid β-protein precursor (APP) and suppresses the intracellular metabolism of APP [1], [6]–[9]. APP is a type I membrane protein, subject to *N*-glycosylation (immature APP/imAPP) in the endoplasmic reticulum (ER) in the early protein secretory pathway, and further subject to *O*-glycosylation in the Golgi compartment in the late secretory pathway [10]. Mature APP (mAPP), possessing both *N*- and *O*-glycans, is subject to consecutive cleavages at the extracellular juxtamembrane region by α- or β-secretase, and at the transmembrane region by γ-secretase in the late secretory pathway [11]. This amyloidogenic cleavage of mAPP by β- and γ-secretases generates neurotoxic amyloid β-protein (Aβ), and the generation and oligomer formation of Aβ are widely believed to be the primary cause of Alzheimer's disease (AD) [12]. Other substrates of γ-secretase such as APP-like proteins and neurexin, but not Notch, also bind to X11s, although the interaction is not restricted to type I membrane proteins [5].

In transgenic mice, overexpressing X11 or X11L, together with the human APP Swedish mutation, resulted in a decrease of cerebral Aβ levels and Aβ plaque formation when compared to mice overexpressing the human APP Swedish mutation alone [13], [14]. Conversely, the amyloidogenic metabolism of endogenous APP and overexpressed human APP, including the generation of Aβ, was enhanced in the brains of X11-deficient, X11L-deficient and X11/X11L doubly-deficient mice [15]–[17]. In the brain, X11s are thought to associate with mAPP outside the detergent-resistant membrane (DRM) or lipid raft, which is rich in active β-secretase (BACE) [18]. It was also reported that dysfunctional X11s in X11s knock-out mice facilitated the entry of mAPP into the DRM and enhanced the cleavage of uncomplexed mAPP by BACE [16].

In addition to suppression of the amyloidogenic cleavage of mAPP by direct binding between the PTB/PI domain of X11s and the 681-GYENPTY-687 motif in the APP cytoplasmic region [19], [20], X11s show other regulations in APP metabolism. Indeed, cells overexpressing X11s showed intracellular imAPP accumulation, which means a suppression of APP maturation by invalidation of *O*-glycosylation [1]. This resulted in a reduction in the secretion of the *N*-terminal APP fragment, sAPPα/β; in the generation of the C-terminal APP fragment, APP CTFα/β (generated from the primary cleavage of APP by α- or β-secretase); and in the secretion of Aβ, generated by the intramembranous γ-cleavage of CTFβ [1], [6], [7]. Thus, the intracellular trafficking of APP, including the APP maturation steps regulated by X11s, is closely associated with APP metabolism [11], [19].

In this study, we performed a functional dissection of X11L, which revealed that: (1) the C-terminal PDZ domains of X11L regulate the passage of APP into early secretory pathway without the direct-binding to APP and consequently regulate overall APP metabolism including Aβ generation, and (2) the imAPP accumulated in early secretory pathway is subject to transport to plasma membrane by unidentified way.

## Results

### Identification of X11L domain involved in the suppression of APP maturation

**Figure 1:**
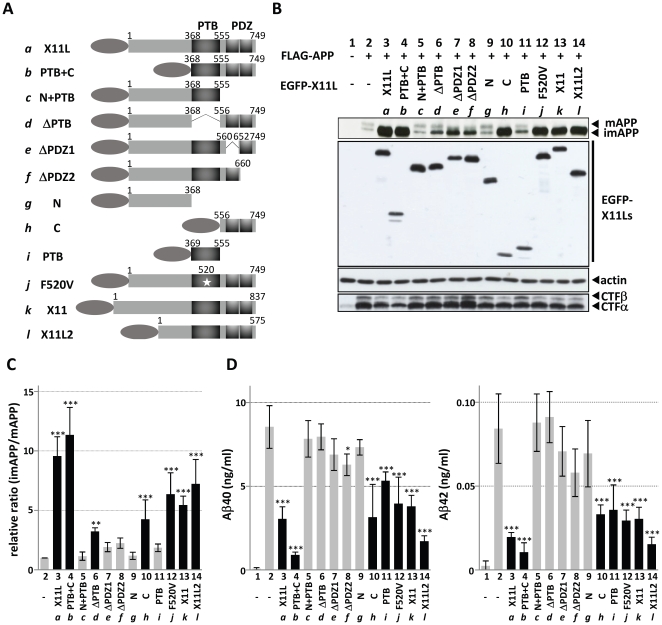
Suppression of APP maturation and proteolytic metabolism by the PDZ domains of X11 proteins. (**A**) Schematic representation of the structures of amino-terminal tagged X11L proteins. *a*: human X11L, *b*: X11L-PTB+C (368 to 749), *c*: X11L-N+PTB (1 to 555), *d*: X11L-ΔPTB (Δ369 to 555), *e*: X11L-ΔPDZ1 (Δ561 to 651), *f*: X11L-ΔPDZ2 (Δ661 to 749), *g*: X11L-N (1 to 368), *h*: X11L-C (556 to 749), *i*: X11L-PTB (369 to 555), *j*: X11L F520V, *k*: human X11, and *l*: human X11L2. PTB, phosphotyrosine binding domain; PDZ, PDZ domain. Oval indicates FLAG (Fig. 2) or EGFP (Fig. 1, 3, 6) tag. Numbers indicate amino acid positions. (**B to D**) Alteration of APP maturation and proteolytic metabolism in cells expressing EGFP-X11L mutant proteins, X11 and X11L2. N2a cells (∼2×10^5^) were transiently transfected with pcDNA3-FLAG-APP695 (0.8 µg) in the presence of various X11L cDNA plasmids, pcDNA3.1-EGFP-X11L and pcDNA3.1-EGFP-X11L2 (*a*: 0.18 µg, *b*: 0.6 µg, *c*: 0.6 µg, *d*: 0.06 µg, *e*: 0.36 µg, *f*: 0.18 µg, *g*: 0.08 µg, *h*: 0.15 µg, *i*: 0.6 µg, *j*: 0.54 µg, *k*: 0.09 µg, and *l*: 0.12 µg). Plasmid amounts were adjusted to produce a similar level of protein expression, and empty vector was added to yield a total of 1.4 µg of plasmid to standardize the amount of plasmid. The cell lysates were analyzed by immunoblotting with an anti-FLAG M2 antibody to detect APP, an anti-EGFP antibody to detect X11L derivatives, an anti-actin antibody to detect actin, and an anti-APP/C antibody to detect APP-CTFα/β (**B**). (**C**) The relative ratio of imAPP/mAPP is quantified. The relative ratio in cells in the absence of X11L was set as 1.0 (column 2). (**D**) Aβ40 and Aβ42 secreted into the medium of N2a cells were quantified with sELISA. Concentrations of Aβ40 (left) and Aβ42 (right) are shown as means ± s.e. (n = 4). Data were analyzed using the Student's *t*-test (*, *P*<0.05; **, *P*<0.01; ***, *P*<0.001). Proteins showing a significant decrease (P<0.01 and P<0.001) in Aβ generation are shown as closed columns.

APP is cleaved by secretases during the late secretory pathway after it has undergone glycosylation; thus, mAPP is indeed the substrate of the secretases and Aβ is generated from mAPP [11], [19]. Previous studies showed that overexpression of X11L suppressed APP maturation and resulted in the accumulation of imAPP in cells [1]. To identify the functional region of X11L involved in the suppression of APP maturation, we prepared various domain-deleted X11L mutants (**Fig. 1A**). N-terminal EGFP-tagged X11L mutants were expressed in N2a cells with FLAG-APP, and the maturation of APP was examined by immunoblotting (**Fig. 1B**). The ability of X11L to suppress the maturation of APP and to accumulate imAPP is indicated as the relative ratio of imAPP to mAPP (imAPP/mAPP) (**Fig. 1C**) in which the ratio of cells with no X11L expression was set as 1.0 (column 2).

Moderate amounts of mAPP was detected along with imAPP in cells in the absence of X11L expression (**Fig. 1B**, lane 2), while the expression of X11L in these cells resulted in the suppression of APP maturation and the significant accumulation of imAPP, which also leads to a decrease in the levels of both CTFα and CTFβ, the products of mAPP cleavage by primary α- or β-secretase (compare lane 2 to lane 3; to indicate CTFβ, a relative darker exposure of film was shown in lower row of **Fig. 1B**).

In response to the expression of the X11L mutants, which showed expression levels almost identical to that of wild-type X11L (some mutants with smaller size, PTB+C, C and PTB, tend to express in lower level slightly), PTB+C (**Fig. 1B**, lane 4; Fig. 1C, column 4) and C (**Fig. 1B**, lane 10; Fig. 1C, column 10) produced a significant accumulation of imAPP, accompanied with a decrease in CTFα and CTFβ (compare lanes 4 and 10 to lane 2 in the lower row of **Fig. 1B**). The data indicate that X11L including the two PDZ domains, PTB+C (column 4), ΔPTB (column 6), C (column 10), and F520V (column 12) mutants along with entire X11L (column 3) showed a significant accumulation of imAPP (**Fig. 1C**), suggesting that the two PDZ domains of X11L are required for the suppression of APP maturation.

**Figure 2:**
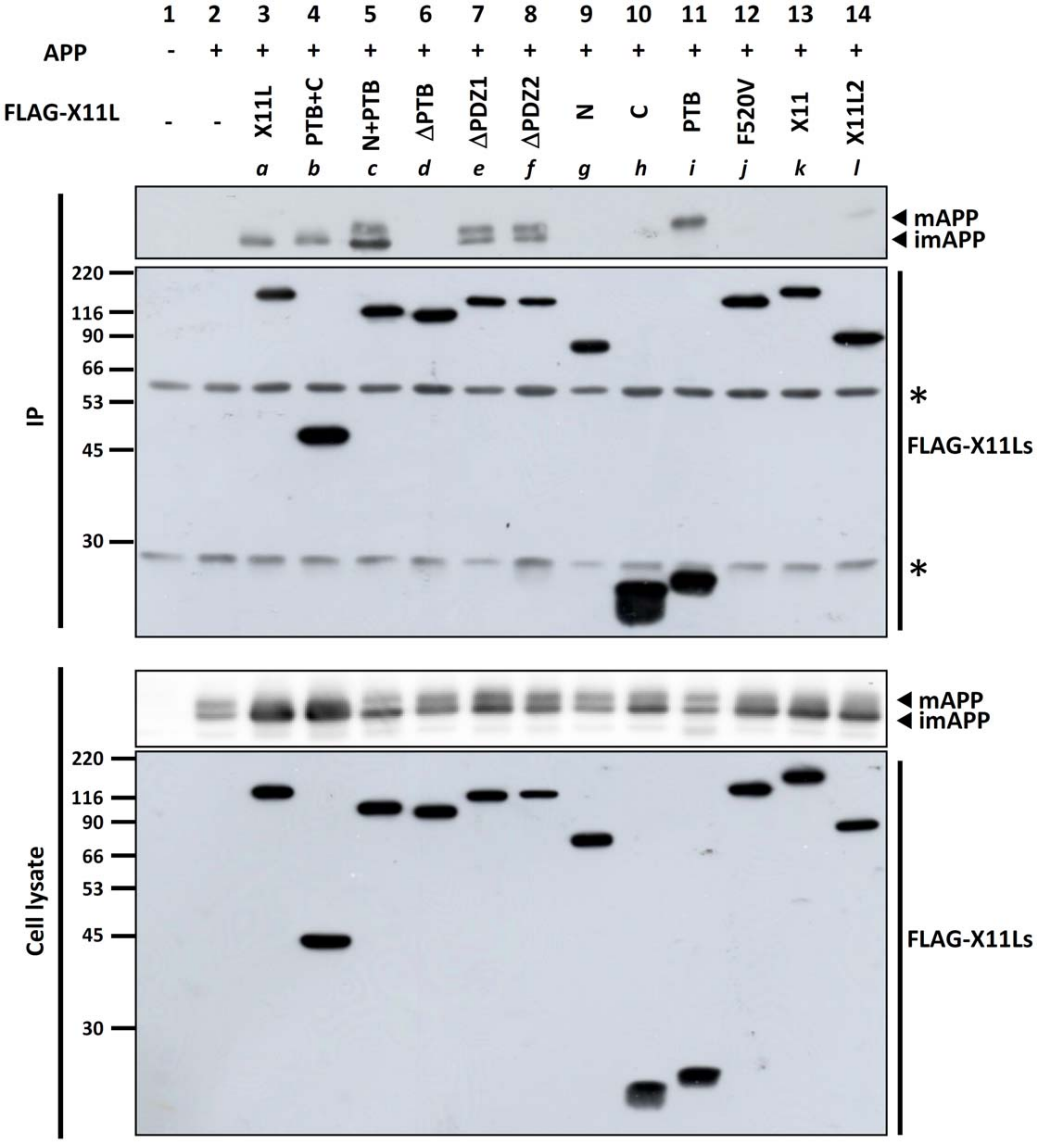
PTB domain-dependent binding of X11L to APP. N2a cells (∼2×10^5^) were transiently transfected with pcDNA3-APP695 (0.6 µg) in the presence of various cDNA plasmids encoding the FLAG-X11L proteins: FLAG-X11 and FLAG-X11L2 (*a*: 0.2 µg, *b*: 0.8 µg, *c*: 0.4 µg, *d*: 0.2 µg, *e*: 0.2 µg, *f*: 0.4 µg, *g*: 0.4 µg, *h*: 0.1 µg, *i*: 0.8 µg, *j*: 0.2 µg, *k*: 0.2 µg, and *l*: 0.2 µg). Plasmid amounts were adjusted to produce a similar level of protein expression, and empty vector was added to yield 1.4 µg of plasmid in total to standardize the plasmid amount. The cell lysates were subjected to immunoprecipitation with an anti-FLAG antibody. The immunoprecipitate (IP) and cell lysate were analyzed by immunoblot analysis with anti-FLAG or anti-APP/C antibodies. Lanes *a* to *l* correspond to the protein constructs described in Figure 1A. Numbers indicate protein molecular weight standards (kDa). Asterisks indicate non-specific, immuno-reaction products. Association of X11s with APP is also shown in Figure S1.

Because it has been widely accepted that X11s can regulate APP metabolism by a direct binding to APP through its PTB domain [1], [6]–[9], we also verified whether the association of APP with X11L is required for the accumulation of imAPP. It has already been known that X11L lacking APP-binding PTB domain (ΔPTB; lane 6 in **Fig. 1B**) and X11L possessing an amino acid substitution at Phe520 (F520V; lane 12 in **Fig. 1B**) don't interact with APP [21]. The interaction of APP with the X11L mutants used in this study was examined by coimmunoprecipitation (**Fig. 2**), which reconfirmed that the PTB domain of X11L binds to APP as described previously [1]. Although ΔPTB and F520V mutants had an effect on imAPP accumulation and caused a decrease in CTFα and CTFβ (**Fig. 1B and C**), we confirmed that both mutants did not associate with APP (**Fig. 2**, lanes 6 and 12). Therefore, we concluded that the C-terminal region, which includes the PDZ domains, is involved in the accumulation of imAPP, without being bound to APP directly. This regulation by X11L in APP metabolism, therefore, is independent of the suppression of amyloidogenic cleavage of mAPP by X11L which is widely known as X11s function in the regulation of APP metabolism [5], [16], [22].

The PTB+C mutant (**Fig. 1B**, lane 4; **Fig. 1C**, column 4) strongly suppressed the maturation of APP compared to the C mutant, which contained only the C-terminal region (**Fig. 1B**, lane 10; **Fig. 1C**, column 10). Attachment of the N-terminal PTB domain to the C region may have stabilized the conformation of the C region included the two PDZ domains, because X11, a member of X11 family proteins, is reported to form the closed conformation of the PDZ domains [23]. The ΔPTB mutant, possessing an intact C-terminal region also showed a weak activity in imAPP accumulation (**Fig. 1B**, lane 6; **Fig. 1C**, column 6). Lack of a central PTB domain may make difficult to preserve functional C-terminal structure.

X11 and X11L2 expression also showed effects comparable to those of X11L (**Fig. 1B**, lanes 13 and 14, and **Fig. 1C**, columns 13 and 14), regardless of their weaker binding to APP compared to X11L (compare lane 3 with lanes 13 and 14 in **Fig. 2**, and see **Fig. S1** for confirmation of X11s binding to APP). This result suggests that all members of X11s has ability to accumulate imAPP and also supports that the association strength between APP and the X11s is not involved in the accumulation of imAPP.

X11L mutants carrying the two PDZ domains but lacking APP-binding ability also showed a significant decrease in the secretion of both Aβ40 and Aβ42 derived from mAPP (**Fig. 1D**: C, column 10; F520V, column 12), as did entire X11s (X11L, column 3; X11, column 13; X11L2, column 14) and X11L mutants containing PTB domain (PTB+C, column 4; PTB, column11). In contrast to these, ΔPDZ1 (column 7) did not suppress Aβ generation and ΔPDZ2 (column 8) showed very weak suppression of Aβ40 alone. The observations suggest that existence of both PDZ domains is significant for the Aβ suppression, which is thought to dependent on the suppression of APP maturation. The ΔPTB mutant, in spite of the preservation of intact PDZ domains, had no effect on Aβ suppression. This may reflect to a weak activity in imAPP accumulation (**Fig. 1C and D**, column 6), as described above, probably by the conformation of C-terminal structure. In contrast to this, PTB alone (**Fig. 1D**, column 11) had an effect on the suppression of Aβ generation without significant accumulation of imAPP (**Fig. 1C**, column 11). This PTB fragment may be relatively free to localize in cytoplasm and can associate predominantly to mAPP (**Fig. 2**, lane 11), when compared to other PTB containing X11L peptide (**Fig. 2**, lanes 4, 5, 7 and 8), and is thought to suppress the amyloidogenic processing of mAPP as described previously [16].

Therefore, strong effect of X11L in the suppression of Aβ generation is composed of, at least, two functions; one is the suppression of amyloidogenic processing of mAPP in late protein secretory pathway [16] in which X11L binds to mAPP through the PTB domain, and another is the regulation of APP maturation in early protein secretary pathway, where the two PDZ domains of X11L function to imAPP independent of the direct binding.

### Appearance of imAPP on plasma membrane of cells expressing X11s

**Figure 3:**
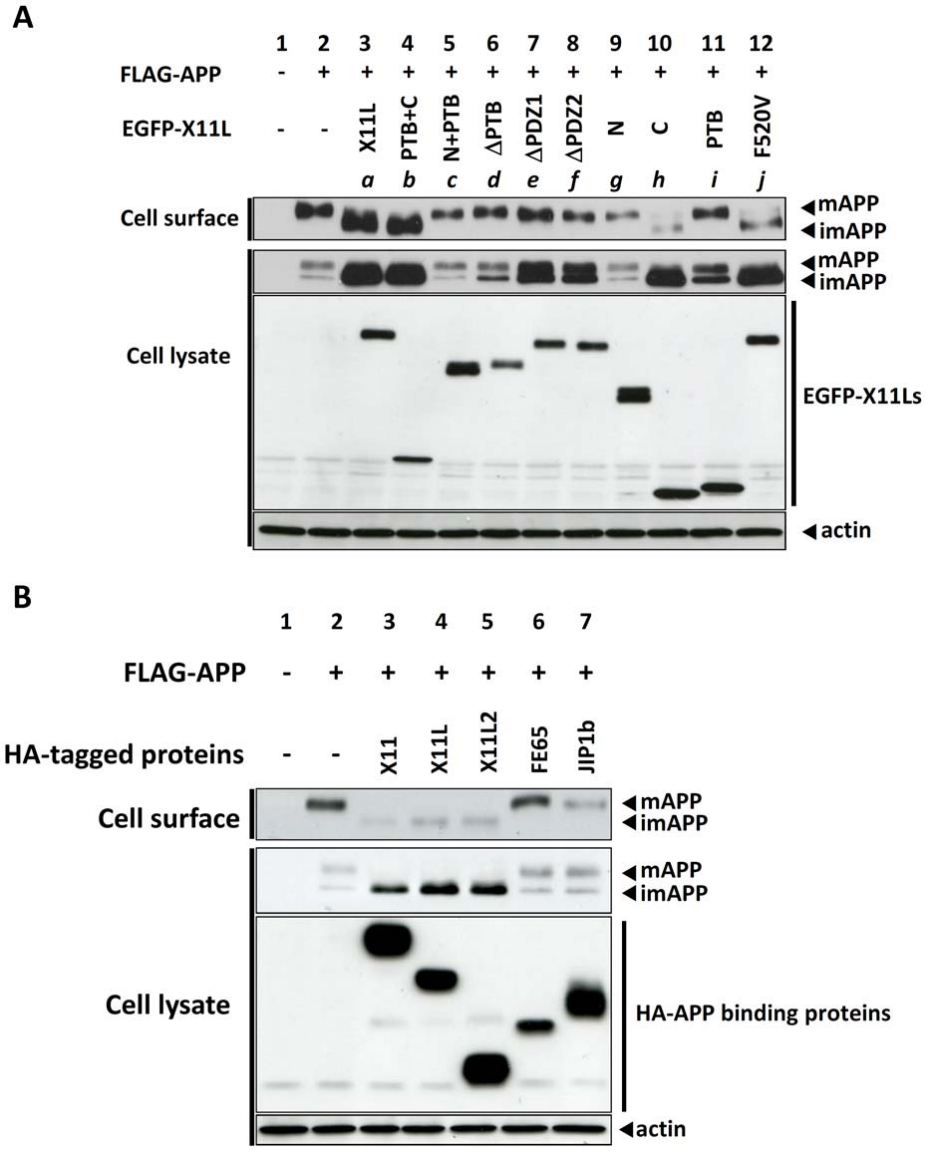
Cell surface expression of imAPP induced by X11L mutant proteins. (**A**) Effect of X11L mutant proteins. N2a cells (∼2×10^5^) were transiently transfected with pcDNA3-FLAG-APP (0.8 µg) in the presence of various cDNA plasmids encoding EGFP-X11L proteins (*a*: 0.18 µg, *b*: 0.6 µg, *c*: 0.6 µg, *d*: 0.06 µg, *e*: 0.36 µg, *f*: 0.18 µg, *g*: 0.08 µg, *h*: 0.15 µg, *i*: 0.6 µg, and *j*: 0.54 µg). To standardize the plasmid amount, empty vector was added to yield 1.4 µg of plasmid in total. Cells were labeled with sulfo-NHS-LC-biotin and NeutrAvidin was used to collect the biotinylated proteins. The cell lysates and biotinylated proteins (Cell surface) were analyzed by immunoblotting with an anti-FLAG antibody to detect APP, an anti-EGFP antibody to detect X11L proteins, and an anti-actin antibody to detect actin. Lanes *a* to *j* correspond to the constructs described in Figure 1A. (**B**) Specificity of X11s function. N2a cells (∼2×10^5^) were transiently cotransfected with pcDNA3-FLAG-APP695 (0.6 µg) and 0.2 µg of the following plasmids: pcDNA3.1-HA-X11 (lane 3), pcDNA3.1-HA-X11L (lane 4), pcDNA3.1-HA-X11L2 (lane 5), pcDNA3.1-HA-FE65 (lane 6), or pcDNA3.1-HA-JIP1b (lane 7). Cells were labeled with sulfo-NHS-LC-biotin and NeutrAvidin was used to collect biotinylated proteins. The cell lysate and biotinylated proteins (Cell surface) were analyzed by immunoblotting with an anti-FLAG antibody to detect APP, an anti-HA antibody to detect HA-binding proteins, and an anti-actin antibody to detect actin.

Next, we explored the localization of imAPP accumulated in cells expressing X11L. We considered that the imAPP dominantly accumulated in ER and/or early Golgi as a matter of course. Contrary to our expectations, we found that some of imAPP accumulated in cells appeared on the plasma membrane without *O*-glycosylation (**Fig. 3A**). Proteins exposed on the outer surface of cells expressing FLAG-APP in the presence of various EGFP-X11L proteins were biotinylated, recovered by NeutrAvidin beads from cell lysates, and subjected to immunoblotting with an anti-FLAG antibody. Usually only mAPP is detected on the plasma membrane (**Fig. 3A**, lane 2) because imAPP is localized at the early protein secretory pathway [11], [19]. Nevertheless, imAPP was detected on the plasma membrane of cells expressing X11L (lane 3), reproducibly. In this study, actin (cytoplasmic protein), calnexin (ER protein) and EGFP-X11L were not detected in biotinylated cell-surface samples (**Fig. S2**), indicating that only cell-surface proteins were biotinylated.

Cell surface-localized imAPP was also observed in cells expressing the PTB+C mutant (**Fig. 3A**, lane 4), and a moderate level of imAPP was detected on the surface of cells expressing C (**Fig. 3A**, lane 10) and F520V (lane 12). The mutants lacking either or both PDZ domains (N+PTB, lane 5; ΔPDZ1, lane 7; ΔPDZ2, lane 8; N, lane 9; PTB, lane 11) had no effect on the presence of imAPP on the plasma membrane. One mutant ΔPTB, possessing an intact C-terminal region, also had no effect (lane 6) which may be due to a weak activity in imAPP accumulation (**Fig. 1B**, lane 6; **Fig. 1C**, column 6; **Fig. 3A**, lane 6 of second row). These observations suggested that some of imAPP accumulated by expression of X11L proteins containing C-terminal PDZ domains was transported to plasma membrane and this activity might tend to be enhanced in the presence of entire PTB domain (compare lane 4 to lane10, and compare lane 3 to lane 12 in **Fig. 3A**).

The observation that imAPP exposed on the plasma membrane of cells that expressed with X11L implies that imAPP, which was not subjected to *O*-glycosylation, might be transported to the plasma membrane by unidentified mechanism, probably differs from the classical Golgi mediated protein secretory pathway. To exclude the possibility that X11L suppressed protein *O*-glycosylation in the Golgi, we examined the expression of the vesicular stomatitis virus G protein (VSVG), a protein subjected to *O*-glycosylation [24], and found that *O*-glycosylated VSVG was detected in cells in the presence, and absence, of X11L expression (**Fig. S3**). Although we cannot rule out a possibility that the presence of X11L may specifically interfere with the *O*-glycosylation of imAPP without affecting the *O*-glycosylation of VSVG, this result supports our observation that imAPP was transported to the plasma membrane, by unidentified way, probably without passing Golgi mediated protein secretory pathway.

To reconfirm that protein mobility shift on SDS-PAGE is due to the *O*-glycosylation of APP (this has been demonstrated in our and others' previous works [10], [19], [25]) , APP was recovered from cells with or without X11L expression, after cell surface labeling of proteins with biotin, by immunoprecipitation with anti-APP antibody (**Fig. S4**). The immunoprecipitated APP was treated with endoglycosidase H to remove *N*-glycans, neuraminidase to remove neuraminic acids, and a combination of neuraminidase and *O*-glycanase (*O*-glycans with terminal neuraminic acid show resistance to *O-*glycanase) to remove *O*-glycans [10], and APP exposed on the outer surface of cells was detected by immunoblotting with anti-biotin antibody. The outer surface-localized mAPP of cells without X11L expression moved down to the position of imAPP on SDS-PAGE (**Fig. S4**, left), indicating that *O-*glycans were removed from mAPP. In contrast to this, the outer surface-localized imAPP of cells with X11L expression did not show the mobility shift by enzyme treatment (**Fig. S4**, right), indicating that imAPP, which is not subject to *O-*glycosylation, appeared to the outer surface of cells in the presence of X11L.

This effect is characteristic of X11 family proteins. Other APP-binding proteins, such as FE65 and JIP1b which are also recognize the 681-GYENPTY-687 motif in the APP cytoplasmic region [19], [20], did not accumulate imAPP in cells and appear imAPP on the plasma membrane (**Fig. 3B**).

**Figure 4 pone-0022108-g004:**
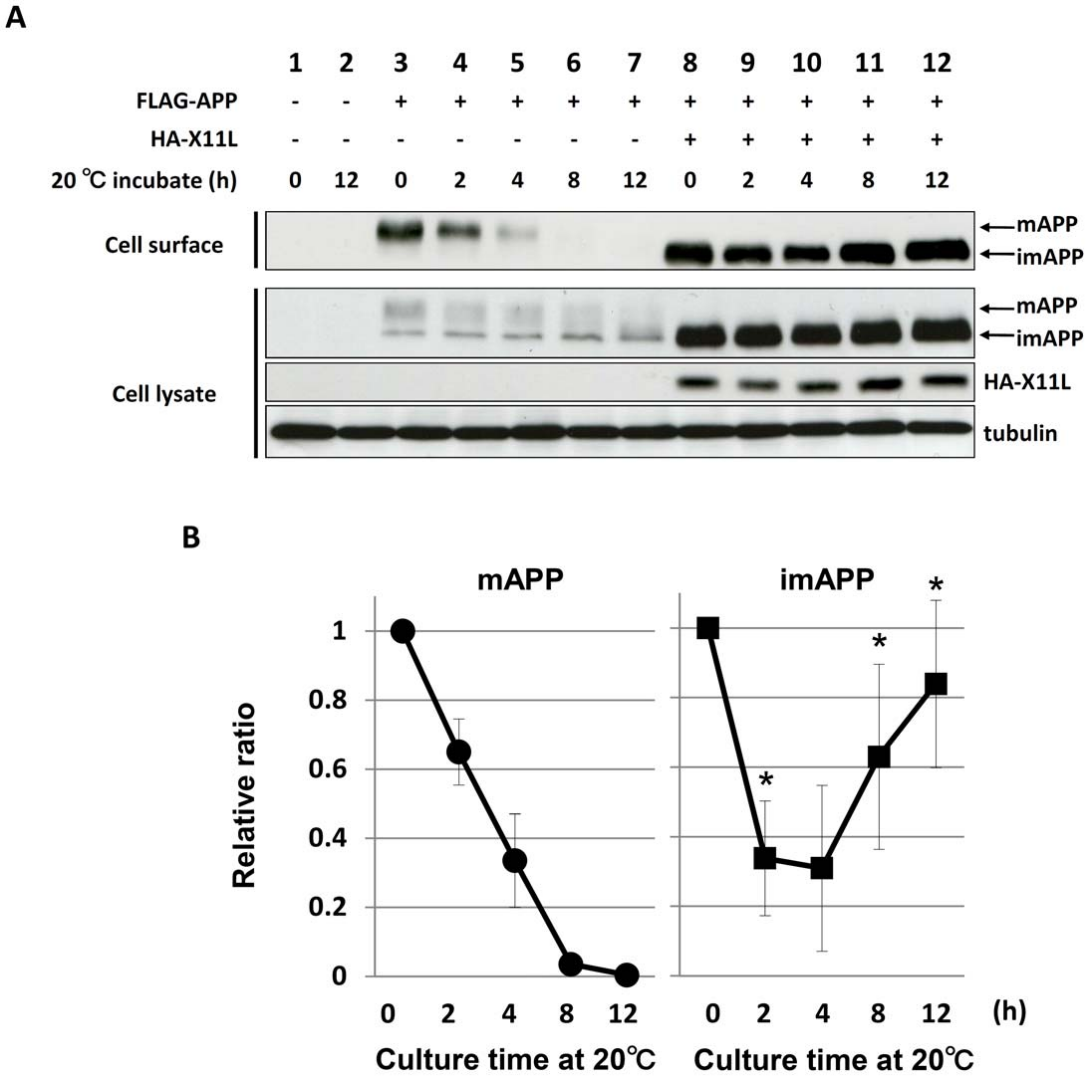
Cell surface appearance of imAPP by X11L expression in cells suffered from reduced temperature. N2a cells (∼1×10^6^) transferred transiently with 0.6 µg of pcDNA3-FLAG-APP with (+) or without (−) 0.2 µg of pcDNA3.1-HA-X11L were cultured for indicated time (h) at 20°C. To standardize the plasmid amount, empty vector was added to yield 0.8 µg of plasmid in total. The cells were labeled with sulfo-NHS-LC-biotin and NeutrAvidin was used to collect biotinylated proteins. (**A**) The cell lysates and biotinylated proteins (Cell surface) were subjected to immunoblot analysis with anti-FLAG antibody to detect APP, anti-HA antibody to detect X11L, and anti-α-tubulin antibody to detect α tubulin. (**B**) The relative ratio of cell surface mAPP in cells without X11L expression (first row of left panel; lanes 3 to 7 in **A**) and that of cell surface imAPP in cells with X11L expression (first row of right panel; lanes 8 to 12 in **A**) are quantified. The relative ratio in cells at time 0 (lanes 3 and 8) was set as 1.0. Data were analyzed using the Student's *t*-test with standard error (n = 4; *, P<0.05).

We further and carefully performed studies to verify the transport of imAPP. It is known that the reduced temperature prevents transfer of membrane glycoproteins to cell surface [26], [27]. Cells with or without expression of X11L were cultured at 20°C for 0 to 12 h and APP exposed on the outer surface of cells was analyzed by immunoblotting along with total cellular APP (**Fig. 4**). In the absence of X11L expression, cell surface-localized mAPP decreased depending on culture time and no surface mAPP was detected at 8 h (**Fig. 4A**, lanes 3 to 7), while in the presence of X11L, cell surface-localized imAPP first decreased by 2–4 h but re-increased at 8 to 12 h (**Fig. 4A**, lanes 8 to 12). The quantitative analysis of APP exposed on the outer surface of cells was also shown in **Fig 4 B** (left, mAPP on cell surface of cells without X11L expression; right, imAPP on cell surface of cells with X11L expression). This study supports our idea that X11L mediates imAPP transfer onto plasma membrane by unidentified mechanism but not through the conventional Golgi mediated secretory pathway.

**Figure 5:**
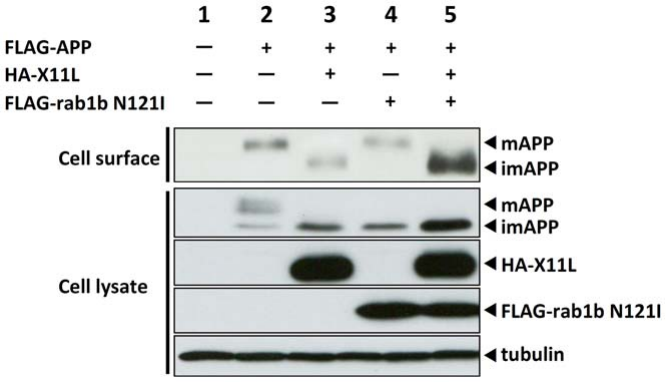
Cell surface appearance of imAPP by X11L expression in cells expressing Rab1b dominat-negative mutant. N2a cells (∼2×10^5^) were transferred transiently with 0.3 µg of pcDNA3-FLAG-APP with (+) or without (−) 0.1 µg of pcDNA3.1-HA-X11L and 0.4 µg of pCA-FLAG-Rab1bN121I. To standardize the plasmid amount, empty vector was added to yield 0.8 µg of plasmid in total. The cells were labeled with sulfo-NHS-LC-biotin and NeutrAvidin was used to collect biotinylated proteins. The cell lysates and biotinylated proteins (Cell surface) were subjected to immunoblot analysis with anti-FLAG antibody to detect APP, anti-HA antibody to detect X11s, and anti-α-tubulin antibody to detect α tubulin.

Rab1 is known as an essential factor required for the ER to Golgi transport [28]. We analyzed the transport of APP from the ER to the plasma membrane using dominant-negative mutants of Rab1a and Rab1b. We explored four dominant-negative mutants, Rab1aS25N (Asn was substituted for Ser25 of Rab1a), Rab1aN124I (Ile was substituted for Asn124), Rab1bS22N (Asn was substituted for Ser 22 of Rab1b) and Rab1bN121I (Ile was substituted for Asn121) in the effect of the accumulation of imAPP (**Fig. S5**). As expected, these dominant-negative Rab1 mutants inhibited the maturation of APP and accumulated imAPP in cells. Because Rab1bN121I mutant was most effective to accumulate imAPP, we further examined whether X11L expression performs the cell surface transport of imAPP which was accumulated in the ER by the expression of Rab1bN121I mutant (**Fig. 5**). In the absence of X11L, mAPP alone appeared on cell surface and the amount was decreased by the expression of Rab1bN121I mutant (**Fig. 5**, compare lane 2 to lane 4 on first row). The expression of Rab1bN121I increased intracellular imAPP (compare lane 4 to lane 2 on second row) but the imAPP did not appear on cell surface (lane 4 on first row). When X11L was expressed with Rab1bN121I, imAPP appeared on the cell surface (lane 5 on first row) along with accumulated intracellular imAPP (lane 5 on second row), indicating that imAPP accumulated in ER under the expression of dominant-negative mutant of Rab1b was transferred to the plasma membrane without passing Rab1b-dependent classical secretory pathway. Taken together, X11L can mediate the transport of imAPP to the plasma membrane by unidentified mechanism, which is different from Golgi mediated protein secretory pathway.

**Figure 6:**
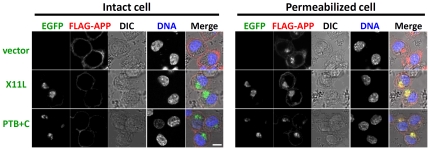
Expression of APP on the outer membrane. N2a cells (∼2×10^4^) were transiently cotransfected with 0.1 µg of pcDNA3-FLAG-APP695 and 0.04 µg of pcDNA3.1-EGFP-X11L, pcDNA3.1-EGFP-PTB+C, or empty vector. The cells were washed with ice-cold PBS, and FLAG-APP present on the cell surface was labeled with anti-FLAG antibody and anti-mouse IgG antibody-conjugated Alexa 546 on ice (Intact cell, ***left panel*** *s*). The same intact cells were permeabilized and intracellular APP was detected with anti-APP/C antibody and anti-rabbit IgG antibody-conjugated Alexa 633 (Permeabilized cell, ***right panels***). The cells were observed using a confocal laser-scanning microscope LSM510 (Carl Zeiss, Oberkochen, Germany). DIC: Differential Interference Contrast microscopy; DNA: nuclear DNA staining with 4′,6-diamino-2-phenylindole (DAPI). Signals are merged in the right-hand rows. Scale bar, 5 µm.

The localization of FLAG-APP in cells expressing X11L and the PTB+C mutant was compared in cells with, or without, permeabilization. Cells expressing X11L and PTB+C showed localization of APP to the plasma membrane when cells were not permeabilized as observed in cells without expression of X11L (**Fig. 6**, left [intact cell]; vector, no expression of X11L), regardless of the fact that almost no mAPP was found in cells expressing X11L and PTB+C (**Fig. 3A**, lanes 3 and 4). No intracellular-staining of FLAG-APP was observed in intact cells. In contrast to this, FLAG-APP was detected both at perinuclear region and to plasma membrane of permeabilized cells (**Fig. 6**, right [permeabilized cell]. This result confirmed that the periplasmic staining of FLAG-APP observed in intact cells was due to signal of imAPP present on the cell surface (**Fig. 3A**). The imAPP exposed on the plasma membrane may be not a result of membrane fusion between the ER and the plasma membrane in response to X11L expression or an observation of ER membrane fragments, because the ER-resident transmembrane protein, calnexin, was not detected on the plasma membrane of cells expressing X11L (**Fig. S2**). These results (**Fig. 6**), together with the biochemical analyses (**Figs. 3** **to** [Fig pone-0022108-g004] **5**), supported our idea that X11L has the ability to transport imAPP, which was accumulated in the early secretory pathway such as ER, to the plasma membrane.

**Figure 7:**
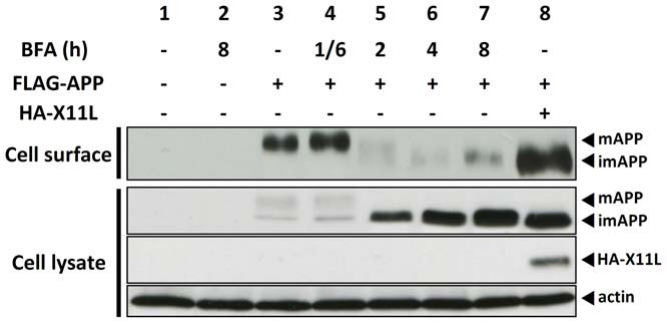
imAPP transport to plasma membrane in cells treated with Brefeldin A. N2a cells (∼2×10^5^) were transiently transfected with pcDNA3.1-FLAG-APP (0.6 µg) in the presence (lane 8) or absence (lanes 3 to 7) of pcDNA3.1-HA-X11L (0.2 µg). To standardize the plasmid amount, empty vector was added to yield 0.8 µg of plasmid in total. Cells were treated with brefeldin A (BFA, 10 µg/ml) for 10 min (1/6 h, lane 4), 2 h (lane 5), 4 h (lane 6) and 8 h (lanes 2 and 7), or without reagent (−, lanes 1, 3, 8). Cells were labeled with sulfo-NHS-LC-biotin and NeutrAvidin was used to collect the biotinylated proteins (Cell surface). The cell lysates and biotinylated proteins were subjected to immunoblot analysis with anti-FLAG antibody to detect APP, anti-HA antibody to detect X11L, and anti-actin antibody to detect actin.

To confirm furthermore that this unidentified pathway that was capable of transporting imAPP to the plasma membrane was not an artificial event due to the exogenous expression of X11L, we explored whether cells also utilized this pathway when APP maturation was suppressed, without the exogenous expression of X11L. Treatment of cells with brefeldin A (BFA) inhibits the transport of secretory/membrane proteins from the ER to the Golgi. In fact, cells treated with BFA showed a remarkable increase in imAPP at 2 hr (**Fig. 7**, lane 5 in second row), and mAPP completely disappeared from the cell surface at 2 h (**Fig. 7**, lane 5 in first row). Interestingly, imAPP appeared on the outer cell surface at 8 h of BFA treatment (lane 7 in first row), which was weak but similar to cells expressing X11L (lane 8 in first row). These observations indicated that cells may naturally possess a secretory pathway that transports imAPP from the ER to the plasma membrane; however, this unidentified pathway may be generally quiescent. When the Golgi mediated protein secretory pathway is subject to malfunction, this pathway, in which X11s is expected to play an important role, may help to dispose of some of the accumulated protein(s). Indeed, in Fig. 7, endogenous X11 proteins, including the ubiquitously expressed X11L2, may function to transport imAPP to the plasma membrane of cells without the exogenous expression of X11L.

## Discussion

The X11 family proteins X11, X11L and X11L2 are adaptor proteins, which contain a divergent N-terminal region that binds synaptic proteins such as Munc-18 and an evolutionarily conserved C-terminal region with a phosphotyrosine binding/phosphotyrosine interaction (PTB/PI) domain that associates with various proteins such as APP, Alcadein and ADP-ribosylation factor (Arf) and two PDZ domains that interact with proteins such as KIF17 and NF-κB [1], [29]–[34]. Cell activities such as intracellular protein transport, presynaptic neurotransmitter release and protein metabolism are regulated through protein-protein interactions mediated by members of the X11 family [5], [20], [35].

In this study, we have focused on the novel role of X11s in the intracellular transport of APP, because Aβ, which is a major factor in the pathogenesis of Alzheimer's disease [12], is generated from the proteolytic cleavages of APP during protein secretory pathway [11]. Among the X11 proteins, X11L binds more strongly to APP than either X11 or X11L2 (**Fig. 2** **and Fig. S1**), and in brain, X11L is expressed more widely than X11, and colocalizes with the expression of APP [16]. Furthermore, X11L-deficient mice showed a remarkable increase in the amyloidogenic processing of endogenous mAPP, including Aβ generation, in the brain compared to X11-deficient mice, which indicated that X11L contributes to the regulation of APP metabolism along with the protein transport in cells to a greater extent than does X11 [15], [16].

At least two independent mechanisms are possible in the suppression of APP metabolism by X11s. One of the mechanisms through which X11s regulate APP metabolism is the suppression of mAPP translocation into the detergent-resistant membrane region (DRM) or lipid raft, where β-secretase is active, by the direct binding of X11s to mAPP. This direct binding by X11s suppresses the amyloidogenic cleavage of APP by β-secretase [16], [22], [35]. X11s also bind to γ-secretase and directly suppress the γ-site cleavage of mAPP [36], [37]. In either situation, the metabolic suppression of mAPP by direct binding to X11s occurs during the late protein secretory pathway. In addition to the suppression of mAPP cleavages, X11s expression in cells induces an overall metabolic stabilization of APP, in other words, X11s induce intracellular accumulation of imAPP, which leads to a consequent decrease in Aβ generation, but the detailed mechanism was still unclear [1], [6]–[9] when compared to X11L function to prevent amyloidogenic cleavage of mAPP [16], [22].

In this study, we analyzed the mechanism of imAPP accumulation in cells with expression of X11L. Our results revealed that the C-terminal PDZ domains of X11L are required for the suppression of APP maturation and the accumulation of imAPP; however, the PTB/PI domain, required for APP-binding, was independent of this activity. Although the molecular mechanism by which X11L suppresses the maturation of imAPP is still under consideration, X11s may be a component to regulate protein transport between early and late Golgi, or the ER and early Golgi.

Another APP-binding protein Numb was reported to accumulate mAPP, but not imAPP, into the early endosomal and recycling compartments of cells [38]. The orchestrated functions of such adaptor proteins to APP in the respective stages of protein secretory pathway may play an important role in the intracellular transport and metabolism of APP.

We found that some of imAPP accumulated in the early secretory pathway by expression of X11L appeared on the cell surface plasma membrane without *O*-glycosylation. The C-terminal PDZ domains of X11L were required for this activity. Although the presence of the PTB/PI domain along with the PDZ domains seemed to enhance the appearance of imAPP on the plasma membrane, the interaction of imAPP with X11L *via* the PTB/PI domain is not involved in the imAPP transport. This imAPP transport to the plasma membrane was verified by three independent procedures; (i) when cells were cultured at 20°C which blocks the secretoty pathway at the late Golgi, cellular imAPP accumulated and the imAPP was appeared on the plasma membrane in the presence of X11L, (ii) when Rab1 dominant-negative mutant which blocks the transport of proteins from the ER to the Golgi were expressed in cells, the accumulated intracellular imAPP appeared on the plasma membrane by expression of X11L, and (iii) when brefeldin A blocked the transport of proteins from the ER to the Golgi, imAPP appeared on plasma membrane in cells, without exogenous expression of X11L.

Taken together, excess X11s suppress the maturation of APP by preventing the imAPP transport into the late secretory pathway, but X11s also show their function to transport the imAPP accumulated in the early secretory pathway to the plasma membrane. Cells are likely to possess this unidentified pathway to transport proteins accumulated in the ER to the plasma membrane, as can be observed in a study with BFA (**Fig. 7**), which may be mediated by X11s, but this pathway must be very slender when classical Golgi-mediated pathway has functioned in healthy cells.

The idea that other membrane and secretory proteins, which are not involved in APP or X11s, may be transported to the plasma membrane from the ER, when proteins are accumulated in early secretory pathway by some reasons, is an interesting one. APP works as a vesicular cargo receptors for the motor protein kinesin-1 [39], [40] Therefore, APP vesicles may contain various types of membrane-associated and/or secretory proteins. Thus, such proteins may also be transported to the plasma membrane when the proteins are unusually accumulated in the early secretory pathway.

In any case, our observations suggest that the existence of an unidentified protein trafficking pathway at least for APP, in which X11s are likely to involve in the regulation of imAPP accumulation and transport. Such novel X11s function, together with the known X11s function of the regulation of APP processing by directly binding to APP, controls APP metabolism through the regulation of APP trafficking. Therefore, understanding for both roles of X11s in APP metabolism and transport should shed light on the molecular mechanism of Aβ generation in Alzheimer's disease.

## Materials and Methods

### Plasmids

The cDNA constructs pcDNA3-APP695, pcDNA3-FLAG-APP695, pcDNA3.1-FLAG-X11L, pcDNA3.1-FLAG-X11L-PTB+C, pcDNA3.1-FLAG-X11L-N+PTB, pcDNA3.1-FLAG-X11L F520V (Phe to Val mutation at amino acid 520), pcDfNA3.1-HA-X11L, pcDNA3.1-HA-X11, pcDNA3.1-HA-X11L2, pcDNA3.1-HA-FE65, pcDNA3.1-HA-JIP1b, pcDNA3.1-EGFP-X11L, pcDNA3.1-EGFP-X11, and pcDNA3.1-EGFP-X11L2 have been described previously [1], [21], [41]–[44]. The constructs, pcDNA3.1-FLAG-X11L-ΔPTB (deletion of amino acids 369 to 555), pcDNA3.1-FLAG-X11L-ΔPDZ1 (deletion of amino acids 561 to 651), pcDNA3.1-FLAG-X11L-ΔPDZ2 (deletion of amino acids 661 to 749), pcDNA3.1-FLAG-X11L-N (amino-terminal amino acids 1 to 368), pcDNA3.1-FLAG-X11L-C (carboxyl-terminal amino acids 556 to 749), and pcDNA3.1-FLAG-X11L-PTB (amino acids 369 to 555) were generated by PCR using pcDNA3.1-FLAG-X11L as the template. The fragments generated were then ligated into pcDNA3.1-FLAG-X11L at the *Nhe*I*/Xho*I site instead of the X11L sequence. The constructs pcDNA3.1-EGFP-X11L-PTB+C, pcDNA3.1-EGFP-X11L-N+PTB, pcDNA3.1-EGFP-X11L F520V, pcDNA3.1-EGFP-X11L-ΔPTB, pcDNA3.1-EGFP-X11L-ΔPDZ1, pcDNA3.1-EGFP-X11L-ΔPDZ2, pcDNA3.1-EGFP-X11L-N, pcDNA3.1-EGFP-X11L-C, and pcDNA3.1-FLAG-X11L-PTB were generated by replacing the FLAG-tag with EGFP. The construct pcDNA3.1-VSV-G-ts045-GFP was kindly gifted from Dr. J. Lippincott-Schwartz [24]. The constructs pCA-FLAG-Rab1aN124I, pCA-FLAG-Rab1aS25N, pCA-FLAG-Rab1bN121I and pCA-FLAG-Rab1bS22N were kindly gifted from D. Y. Kawaoka [45].

### Antibodies

The commercially available antibodies used in this study were purchased as follows: mouse monoclonal anti-FLAG antibody (M2, Sigma-Aldrich, St Louis, MO, USA), anti-HA (12CA5, Roche Diagnostics, Mannheim, Germany), anti-GFP (1E4, Medical & Biological Laboratories/MBL, Nagoya, Japan), anti-GM130 and anti-syntaxin 6 (BD Biosciences, Franklin Lakes, NJ, USA), anti-actin (MAB1501, Chemicon/Millipore, Billeria, MA, USA), anti-α-tubulin (sc-32293, SANTA CRUZ Biotech., Santa Cruz CA, USA) and anti-Aβ (82E1, Immuno-Biological Laboratories/IBL, Fujioka, Japan); rabbit polyclonal anti-APP C-terminal APP/C (A8717, Sigma-Aldrich), and anti-calnexin (Stressgen, Ann Arbor, MI, USA); goat polyclonal anti-rabbit and anti-mouse immunoglobulin antibodies conjugated to horseradish peroxidase (GE Healthcare Life Sciences, Little Chalfont, UK); and goat anti-rabbit and horse anti-mouse immunoglobulin antibodies conjugated to Alexa 546 or Alexa 633 (Molecular probes/Invitrogen, Carsbad, CA, USA).

### Cell culture and expression of proteins

Mouse neuroblastoma cell line Neuro2a (N2a) cells were cultured in Dulbecco's Modified Eagle's Medium (DMEM) supplemented with 10% (v/v) heat-inactivated fetal bovine serum (FBS). To express proteins, approximately (∼) 2×10^5^ cells for a 12-well dish and ∼1×10^6^ cells for a six-well dish were transiently transfected with 0.3 to 1 µg of the plasmids indicated using LipofectAMINE 2000 (Invitrogen, Carlsbad, CA, USA), according to the manufacturer's protocol. The cells were cultured for 32 to 36 h in DMEM containing 10% (v/v) FBS.

### Quantification of Aβ40 and Aβ42

N2a cells (∼1×10^6^) were transiently transfected with pcDNA3-FLAG-APP695 and the plasmid indicated, and then cultured for 32 to 48 h. Aβ40 and Aβ42, secreted into the medium, were quantified using a sandwich enzyme-linked immunosorbent assay (sELISA) as described previously [46]. Briefly, the wells of a 96-well plate were coated with Aβ40 (4D1) or Aβ42 (4D8) end-specific monoclonal antibodies (0.3 µg of IgG in PBS), washed with PBS containing 0.05% (v/v) Tween 20 (PBST), blocked with bovine serum albumin (BSA, 3% [w/v] in PBS), and washed with PBST [10]. Then, a sample (100 µl), suitably diluted with PBST containing 1% (w/v) BSA (dilution buffer), was incubated together with a standard amount of synthetic Aβ1-40 or Aβ1-42 peptide. After washing, the wells were treated with biotinylated 82E1 [47], washed, and incubated with 100 µl of a streptavidin-horseradish peroxidase complex (1∶2,000 dilution; RPN1051, Amersham Pharmacia Biotech). The plates were washed again, and 100 µl of 2,2′-azino-bis (3-ethylbenzothiazoline-6-sulfonia acid) or ABTS solution (KPL 5062-01, Kirkegaard & Perry Laboratories Inc., Gaithersburg, MD) was added to the wells. The plates were incubated at room temperature and the absorbance was measured at 415 nm.

### Co-immunoprecipitation and biotinylation of cell surface proteins

N2a cells (∼1×10^6^) were transiently transfected with the plasmid indicated as described above. Cells were harvested and lysed in HBS-T lysis buffer (10 mM HEPES [pH 7.6] containing 150 mM NaCl, 5 mM EDTA, 0.5% [v/v] Triton X-100, 5 µg/ml chymostatin, 5 µg/ml leupeptin, and 5 µg/ml pepstatin A) and centrifuged at 10,000× g for 10 min. The resulting supernatants were incubated with the antibodies indicated at 4°C for 2 h. The immunocomplex was recovered using protein G-Sepharose beads (GE Healthcare Life Sciences), and the proteins were analyzed by immunoblotting with the antibodies indicated.

Cell surface proteins were biotinylated with EZ-Link Sulfo-NHS-LC-Biotin (Thermo Fisher Scientific, Walthan, MA USA) for 30 min on ice, and the reaction was stopped by washing cells with PBS containing 50 mM glycine. The cells were lysed in HBS-T, and biotinylated proteins were recovered with Immobilized NeutrAvidin Gel (Thermo Scientific) and analyzed by immunoblotting. Actin and calnexin were used as controls to show that the intracellular proteins were not biotinylated.

### Brefeldin A treatment

N2a cells (∼1×10^6^) were transiently transfected with the amount of plasmid indicated as described above and treated with 10 µg/ml of brefeldin A (BFA, Sigma-Aldrich) for 10 minutes, and two, four, or eight hours. Cell surface proteins were biotinylated with EZ-Link Sulfo-NHS-LC-Biotin (Thermo Scientific) for 30 min on ice, and the reaction was stopped by washing cells with PBS containing 50 mM glycine [48]. The cells were lysed in HBS-T, and biotinylated proteins were recovered and analyzed by immunoblotting as described above.

## Supporting Information

Figure S1 **Binding ability of X11 proteins to APP.** N2a cells (∼2×10^5^) were transiently transfected with pcDNA3-FLAG-APP695 (0.6 µg) in the presence (+, 0.2 µg) or absence (−) of pcDNA3.1-HA-X11, pcDNA3.1-HA-X11L and pc DNA3.1-HA-X11L2. To standardize the plasmid amount, empty vector was added to yield 0.8 µg of plasmid in total. The cell lysates were subject to co-immunoprecipitation with anti-FLAG antibody. The immunoprecipitate (IP) and cell lysates were subjected to immunoblot analysis with anti-HA and anti-FLAG antibodies.
(TIFF)

Figure S2 **Specific labeling of cell surface proteins.** N2a cells (∼2×10^5^) were transiently transfected with pcDNA3-FLAG-APP695 (0.6 µg) in the presence (+) or absence (−) of pcDNA3.1-EGFP-X11L (0.2 µg). To standardize the plasmid amount, empty vector was added to yield 0.8 µg of plasmid in total. Cells were labeled with sulfo-NHS-LC-biotin and NeutrAvidin was used to collect biotinylated proteins. The cell lysates and biotinylated proteins (Cell surface) were subjected to immunoblot analysis with an anti-FLAG antibody to detect APP, an anti-EGFP antibody to detect X11L, an anti-calnexin antibody to detect calnexin, and an anti-actin antibody to detect actin.
(TIFF)

Figure S3 **Suppression of** ***O*** **-glycosylation of APP, but not VSVG, by X11L.** (**A**) N2a cells (∼2×10^5^) were transiently transfected with pcDNA3-FLAG-APP695 (0.4 µg) in the presence (+) or absence (−) of pcDNA3.1-HA-X11L (0.4 µg). To standardize the plasmid amounts, empty vector was added to yield 0.8 µg of plasmid in total. The cell lysates were analyzed by immunoblotting with an anti-FLAG (M2) antibody to detect APP and an anti-HA antibody to detect X11L. (**B**) N2a cells (∼2×10^5^) cultured at 32°C or 39.5°C were transiently cotransfected with pcDNA3.1-VSVG-ts045-GFP (0.4 µg) and pcDNA3.1-HA-X11L (0.4 µg) or empty vector (−). The cell lysates were analyzed by immunoblotting with an anti-EGFP antibody to detect VSVG-ts045-GFP and an anti-HA antibody to detect X11L. This temperature sensitive (ts) mutant of the VSVG protein resides in the early secretary pathway at 32°C and is transported into the late secretary pathway where *O*-glycosylation occurs at 39.5°C. The “*mature*” indicates proteins modified with *O*-glycosylation.
(TIFF)

Figure S4 **Characterization of APP exposed on the outer surface of cells.** HEK293 cells were (∼1×10^6^) were transferred transiently with 0.5 µg of pcDNA3-FLAG-APP with (right panel) or without (left panel) pcDNA3-hX11L (Tomita *et al.*, [1999] J. Biol. Chem. 274, 2243–2254). The cell surface proteins were biotynylated and cells were lysed as described in text. Biotinylated APP were treated with the enzymes indicated as descrived previously (Tomita *et al.*, [1998] J. Biol. Chem. 273, 6277–6284) and recovered by immunoprecipitation with anti-APP G369 antibody (Oishi *et al.*, [1997] Mol. Med. 3, 111–123) and the immunoprecipitates were analyzed by immunoblotting with anti-biotin antibody (BN-34, AbcamCambridge, UK). Control, samples treated without enzyme (buffer alone),; Endo H, samples treated with endoglycosidase H; Neu, samples treated with neuraminidase; Neu+O-Gly, samples treated with a combination of neuraminidase and O-glycanase. mAPP, mature APP (*N*- and *O*-glycosylated APP); imAPP, immature APP (*N*-glycosylated APP).
(TIFF)

Figure S5 **Effect of Rab1 dominant-negative forms in intracellular accumulation of imAPP.** N2a cells (∼2×10^5^) were transferred transiently with 0.3 µg of pcDNA3-FLAG-APP with or without (−) 0.3 µg of pCA-FLAG-Rab1aS25N (lane 3), 0.4 µg of pCA-FLAG-Rab1aN124I (lane 4), 0.3 µg of pCA-FLAG-Rab1bS22N (lane 5) or 0.4 µg of pCA-FLAG-Rab1bN121I (lane 6). To standardize the plasmid amount, empty vector was added to yield 0.7 µg of plasmid in total. The cell lysates were subjected to immunoblot analysis with anti-FLAG antibody to detect APP and rab1, and anti-α-tubulin antibody to detect α tubulin. mAPP, mature APP (*N*- and *O*-glycosylated APP); imAPP, immature APP (*N*-glycosylated APP).
(TIFF)
