# Supplementary figures and images for: Intracellular Trafficking of the Amyloid β-Protein Precursor (APP) Regulated by Novel Function of X11-Like
Source: PLoS One. 2011 Jul 19;6(7):e22108. doi: 10.1371/journal.pone.0022108 (PMC3139598; doi:10.1371/journal.pone.0022108)

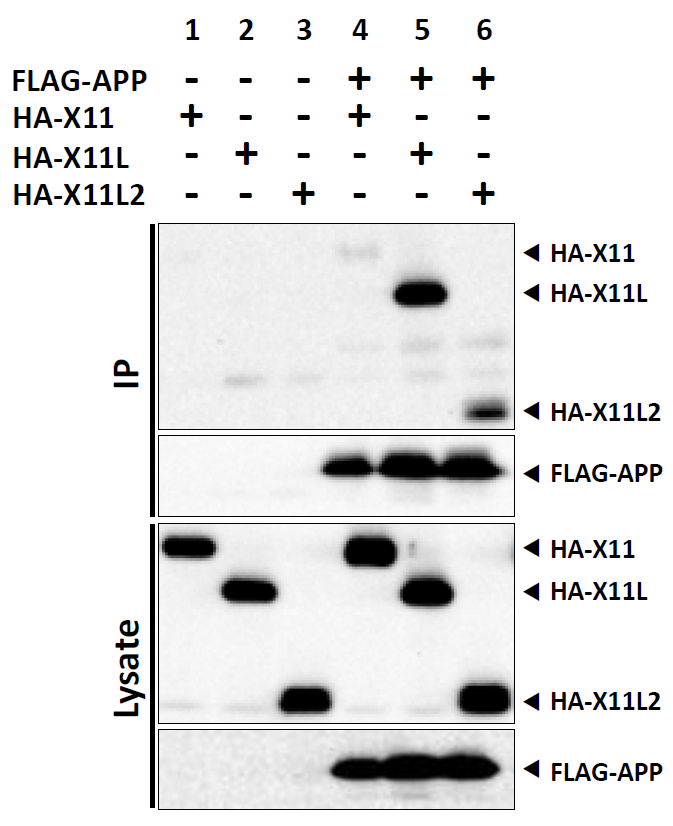

Supplement: Figure S1 — Binding ability of X11 proteins to APP. N2a cells (∼2×10^5^) were transiently transfected with pcDNA3-FLAG-APP695 (0.6 µg) in the presence (+, 0.2 µg) or absence (−) of pcDNA3.1-HA-X11, pcDNA3.1-HA-X11L and pc DNA3.1-HA-X11L2. To standardize the plasmid amount, empty vector was added to yield 0.8 µg of plasmid in total. The cell lysates were subject to co-immunoprecipitation with anti-FLAG antibody. The immunoprecipitate (IP) and cell lysates were subjected to immunoblot analysis with anti-HA and anti-FLAG antibodies. (TIFF) [file pone.0022108.s001.tiff]

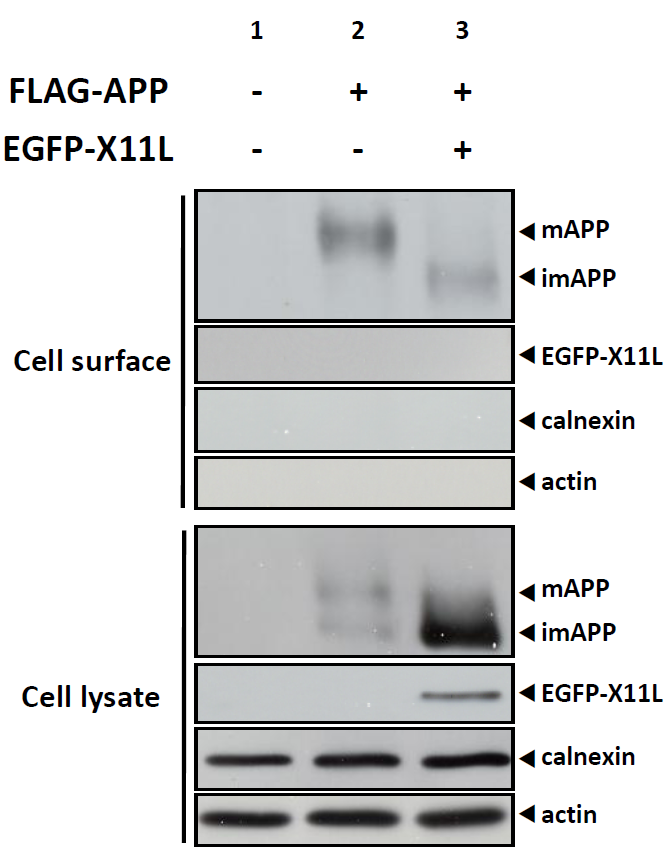

Supplement: Figure S2 — Specific labeling of cell surface proteins. N2a cells (∼2×10^5^) were transiently transfected with pcDNA3-FLAG-APP695 (0.6 µg) in the presence (+) or absence (−) of pcDNA3.1-EGFP-X11L (0.2 µg). To standardize the plasmid amount, empty vector was added to yield 0.8 µg of plasmid in total. Cells were labeled with sulfo-NHS-LC-biotin and NeutrAvidin was used to collect biotinylated proteins. The cell lysates and biotinylated proteins (Cell surface) were subjected to immunoblot analysis with an anti-FLAG antibody to detect APP, an anti-EGFP antibody to detect X11L, an anti-calnexin antibody to detect calnexin, and an anti-actin antibody to detect actin. (TIFF) [file pone.0022108.s002.tiff]

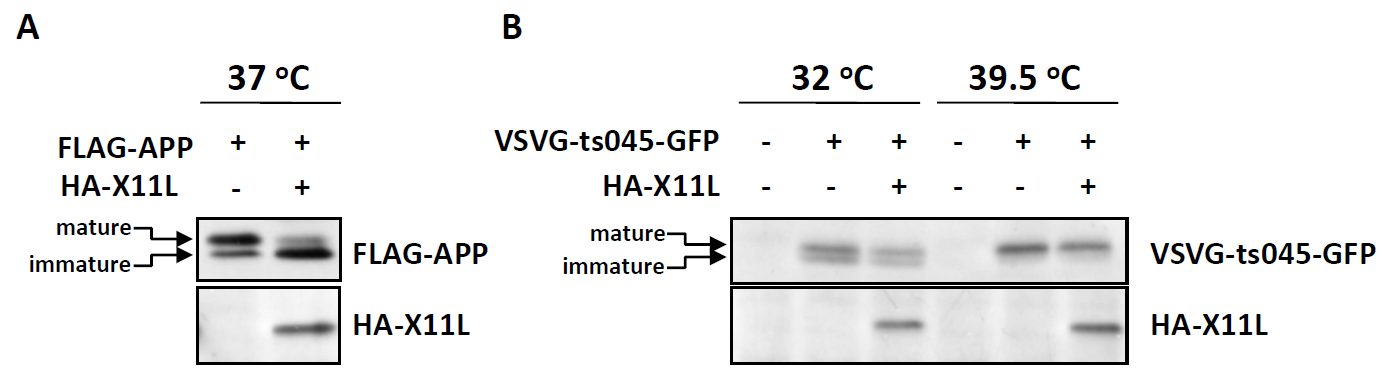

Supplement: Figure S3 — Suppression of O -glycosylation of APP, but not VSVG, by X11L. (A) N2a cells (∼2×10^5^) were transiently transfected with pcDNA3-FLAG-APP695 (0.4 µg) in the presence (+) or absence (−) of pcDNA3.1-HA-X11L (0.4 µg). To standardize the plasmid amounts, empty vector was added to yield 0.8 µg of plasmid in total. The cell lysates were analyzed by immunoblotting with an anti-FLAG (M2) antibody to detect APP and an anti-HA antibody to detect X11L. (B) N2a cells (∼2×10^5^) cultured at 32°C or 39.5°C were transiently cotransfected with pcDNA3.1-VSVG-ts045-GFP (0.4 µg) and pcDNA3.1-HA-X11L (0.4 µg) or empty vector (−). The cell lysates were analyzed by immunoblotting with an anti-EGFP antibody to detect VSVG-ts045-GFP and an anti-HA antibody to detect X11L. This temperature sensitive (ts) mutant of the VSVG protein resides in the early secretary pathway at 32°C and is transported into the late secretary pathway where O-glycosylation occurs at 39.5°C. The “mature” indicates proteins modified with O-glycosylation. (TIFF) [file pone.0022108.s003.tiff]

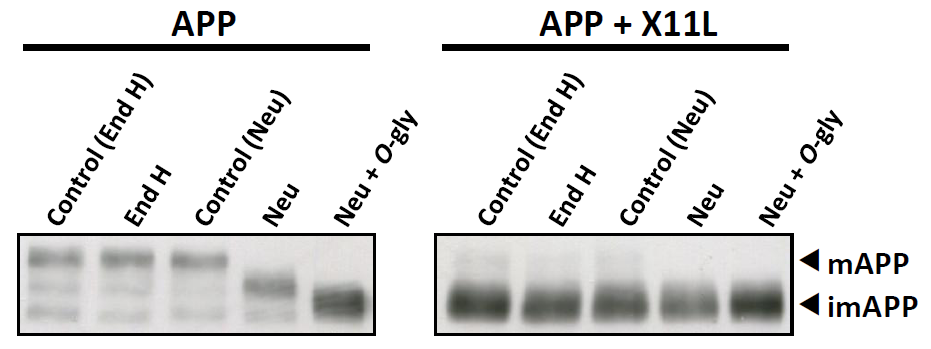

Supplement: Figure S4 — Characterization of APP exposed on the outer surface of cells. HEK293 cells were (∼1×10^6^) were transferred transiently with 0.5 µg of pcDNA3-FLAG-APP with (right panel) or without (left panel) pcDNA3-hX11L (Tomita et al., [1999] J. Biol. Chem. 274, 2243–2254). The cell surface proteins were biotynylated and cells were lysed as described in text. Biotinylated APP were treated with the enzymes indicated as descrived previously (Tomita et al., [1998] J. Biol. Chem. 273, 6277–6284) and recovered by immunoprecipitation with anti-APP G369 antibody (Oishi et al., [1997] Mol. Med. 3, 111–123) and the immunoprecipitates were analyzed by immunoblotting with anti-biotin antibody (BN-34, AbcamCambridge, UK). Control, samples treated without enzyme (buffer alone),; Endo H, samples treated with endoglycosidase H; Neu, samples treated with neuraminidase; Neu+O-Gly, samples treated with a combination of neuraminidase and O-glycanase. mAPP, mature APP (N- and O-glycosylated APP); imAPP, immature APP (N-glycosylated APP). (TIFF) [file pone.0022108.s004.tiff]

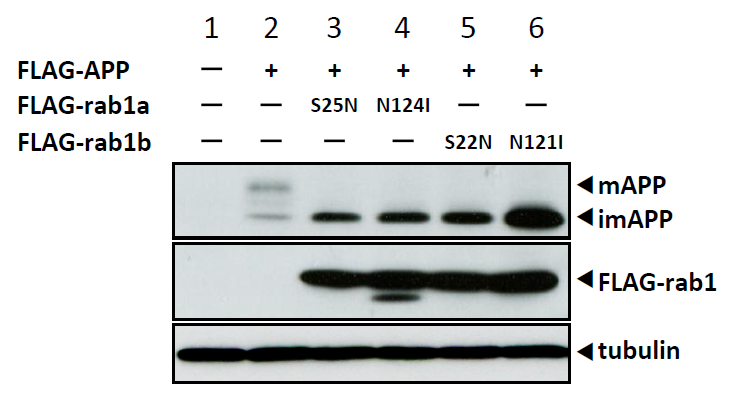

Supplement: Figure S5 — Effect of Rab1 dominant-negative forms in intracellular accumulation of imAPP. N2a cells (∼2×10^5^) were transferred transiently with 0.3 µg of pcDNA3-FLAG-APP with or without (−) 0.3 µg of pCA-FLAG-Rab1aS25N (lane 3), 0.4 µg of pCA-FLAG-Rab1aN124I (lane 4), 0.3 µg of pCA-FLAG-Rab1bS22N (lane 5) or 0.4 µg of pCA-FLAG-Rab1bN121I (lane 6). To standardize the plasmid amount, empty vector was added to yield 0.7 µg of plasmid in total. The cell lysates were subjected to immunoblot analysis with anti-FLAG antibody to detect APP and rab1, and anti-α-tubulin antibody to detect α tubulin. mAPP, mature APP (N- and O-glycosylated APP); imAPP, immature APP (N-glycosylated APP). (TIFF) [file pone.0022108.s005.tiff]
